# Colorful Conductive Threads for Wearable Electronics: Transparent Cu–Ag Nanonets

**DOI:** 10.1002/advs.202201111

**Published:** 2022-07-15

**Authors:** Yan Tang, Bin Guo, Mutya A. Cruz, Han Chen, Qicheng Zhou, Zefeng Lin, Fuchun Xu, Feiya Xu, Xiaohong Chen, Duanjun Cai, Benjamin J. Wiley, Junyong Kang

**Affiliations:** ^1^ Fujian Key Laboratory of Semiconductor Materials and Applications CI center for OSED College of Physical Science and Technology Xiamen University Xiamen 361005 P. R. China; ^2^ Department of Chemistry Duke University Durham NC 27708‐0354 USA

**Keywords:** conductive threads, Cu–Ag nanowires, temperature sensor, warm fabrics, wearable electronics

## Abstract

Electronic textiles have been regarded as the basic building blocks for constructing a new generation of wearable electronics. However, the electronization of textiles often changes their original properties such as color, softness, glossiness, or flexibility. Here a rapid room‐temperature fabrication method toward conductive colorful threads and fabrics with Ag‐coated Cu (Cu‐Ag) nanonets is demonstrated. Cu–Ag core–shell nanowires are produced through a one‐pot synthesis followed by electroless deposition. According to the balance of draining and entraining forces, a fast dip‐withdraw process in a volatile solution is developed to tightly wrap Cu–Ag nanonets onto the fibers of thread. The modified threads are not only conductive, but they also retain their original features with enhanced mechanical stability and dry‐wash durability. Furthermore, various e‐textile devices are fabricated such as a fabric heater, touch screen gloves, a wearable real‐time temperature sensor, and warm fabrics against infrared thermal dissipation. These high quality and colorful conductive textiles will provide powerful materials for promoting next‐generation applications in wearable electronics.

## Graphical abstract

Various types of colorful threads have been modified into conductive by wrapping with Cu‐Ag nanonets without losing their original features. The conductive threads show excellent mechanical stability, dry‐wash durability and sewability. E‐textile devices such as fabric heater, touch screen gloves, temperature sensing cloth, and warm fabrics are fabricated for smart‐fabric applications.

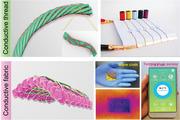

## 1 Introduction

Electronic textiles (e‐textiles) are an emerging development that have great potential for wide applications in next‐generation wearable electronics, which can be used in energy harvesting devices,^[^ ^1^, ^2^ ^]^ various sensing devices,^[^ ^3^, ^4^, ^5^ ^]^ human–machine interfaces,^[^ ^6^, ^7^ ^]^ supercapacitors,^[^ ^8^, ^9^ ^]^ and electromagnetic interference shields.^[^ ^10^, ^11^ ^]^ In the past decades, various efforts have been made to develop electrically conductive textiles and optimize their performance.^[^ ^12^, ^13^, ^14^ ^]^ These techniques can be summarized in three main strategies. Weaving, knitting, and sewing metal wires into textiles is the earliest and most matured method to introduce electrical properties.^[^ ^15^, ^16^ ^]^ Nowadays, commercially available conductive textiles are mostly fabricated using this method. Another technique is to deposit conductive materials such as metal or carbon films on the surfaces of fabrics through methods such as sputtering, electrodeless plating, screen‐printing.^[^ ^17^, ^18^, ^19^, ^20^ ^]^ The third technique (such as dip‐coating, spray‐coating, and drop coating) is creating a coat of conductive materials dispersed in solution, which appears to be an alternative method more suitable for industrialization,^[^ ^21^, ^22^ ^]^ but requires high solubility and dispersivity of the conductive material to achieve high coating uniformity. A variety of conductive materials including metals (Ag, Au, Cu, and Al),^[^ ^23^, ^24^, ^25^ ^]^ carbon‐based materials (graphene, graphene oxide, and carbon nanotube),^[^ ^26^, ^27^, ^28^ ^]^ and conductive polymers (polydimethylsiloxane (PDMS), poly(3,4‐ethylenedioxythiophene):ploy(styrenesulfonate) (PEDOT:PSS), poly(3‐hexylthiophene) (P3HT), and polyaniline)^[^ ^16^, ^29^, ^30^ ^]^ and 2D materials (hexagonal‐boron nitride, transition‐metal dichalcogenides, and MXenes)^[^ ^31^, ^32^, ^33^ ^]^ have been adopted for the preparation of conductive textiles. In principle, these materials have traditional and fundamental limits. Metals are natively heavy and opaque and carbon‐based materials are black. Thus, the fabricated e‐textiles are not able to retain their original color. The stability of conductive polymers still needs to be further improved. Usually, the preparation process of 2D materials are complex. Even with advanced metallic nanowires such as Ag nanowires (NWs), the color of threads, fibers, and textiles totally becomes silvery.^[^ ^34^, ^35^, ^36^, ^37^, ^38^ ^]^ Due to these limitations, highly stable, originally colorful, and light‐weight conductive textiles have not yet been achieved.

From a structural point of view, thread is the building block for textile manufacturing and textiles are usually made of interlaced threads. In principle, successfully producing conductive threads will result in the development of high‐quality e‐textiles. Metal nanowires (NWs) as conductive materials have attracted enormous attention in the fabrication of next‐generation transparent conductors. Moreover, due to the nanoscale diameter of metal NWs, a thin uniform coating can appear transparent. Ag NWs exhibit excellent conductivity and optical transmittance comparable to indium tin oxide (ITO).^[^ ^39^, ^40^ ^]^ The conductivity of Cu NWs is similar to that of Ag NWs,^[^ ^41^ ^]^ but is cheaper and richer in reserves. Both Cu and Ag NWs are mainly synthesized through solution‐based methods and naturally exhibit excellent dispersivity, making them ideal candidates for the solution‐based coating technique.

In this work, we propose a simple and efficient method for synthesizing highly conductive Ag‐coated Cu NWs (Cu–Ag NWs) and develop a fast dip‐wrapping processes to produce various colorful conductive threads for e‐textile applications. Cu NWs were first synthesized in a one‐pot process, followed by electroless deposition step to deposit a thin Ag shell, creating a Cu–Ag core–shell structure. Various types of threads were modified by wrapping with Cu–Ag nanonets to be conductive without losing their original features, and the conductive threads showed excellent mechanical stability, dry‐wash durability, and sewability. E‐textile devices such as a fabric heater, touch screen gloves, a temperature sensing cloth, and warm fabrics were fabricated with outstanding properties. Especially, the temperature sensing cloth can monitor body temperature in real time and only have an error of ±0.3 °C. The warm fabrics can reduce 14.8% power loss owing to high infrared reflectivity of Cu–Ag NWs. The colorful conductive threads and textiles obtained by Cu–Ag nanonets could provide vivid raw materials for the future wearable intelligent fabrics.

## 2 Results and Discussions

**Figure 1:**
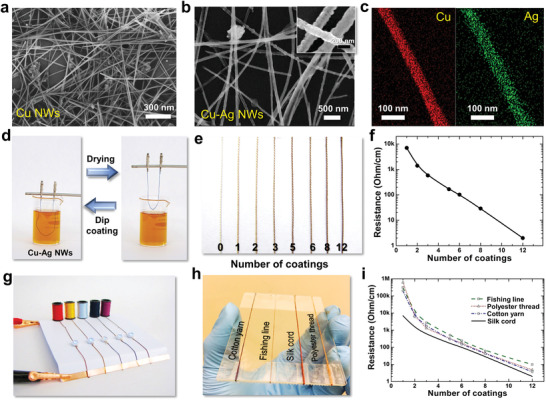
SEM images of Cu NWs a) and Cu–Ag NWs b). The inset of b) is the high magnification SEM image of Cu–Ag NWs. c) EDS elemental mapping images of Cu–Ag NWs. d) The preparation process of conductive thread by dipping Cu–Ag NWs ink solution. e) Photograph of conductive silk cord obtained by different wrapping number of Cu–Ag nanonets. f) Resistance of conductive silk cord as a function of the wrapping number of Cu–Ag nanonets (*n* = 3). g) Colorful conductive threads as conductor to light light‐emitting diode (LED). h) Photograph of different types of threads wrapping with Cu–Ag nanonets. i) Resistance as a function of wrapping number of Cu–Ag nanonets for different types of threads (*n* = 3).

Fine Cu NWs with uniform diameter and high aspect ratio were synthesized through a solution‐based method. However, Cu NWs need further post treatment such as thermal annealing to eliminate or protect from surface oxidation and maintain conductivity.^[^ ^42^ ^]^ To create a protective shell and eliminate the need for laborious annealing, a thin layer of Ag was coated onto the Cu NWs through an electroless deposition technique,^[^ ^43^, ^44^ ^]^ creating a Cu–Ag core–shell structure. **Figure** **1**a,b shows scanning electron microscopy (SEM) images of Cu NWs before and after Ag coating. It can be seen that Ag coating layer makes the surface slightly rougher than that of the pure Cu NWs. Meanwhile, the diameter of Cu–Ag NWs becomes larger. Both transmission electron microscope (TEM) images (Figure S2b, Supporting Information) and Energy Dispersive Spectrometer (EDS)） elemental mapping images (Figure 1c) clearly demonstrate that this method creates a surface layer of Ag that encapsulates the Cu NWs. As shown in Figure S3 (Supporting Information), Fourier Transform infrared spectroscopy (FTIR) spectra revealed that there are no additional formation of chemical bonds between threads and Cu–Ag nanonet. The thickness of the Ag shell can be controlled by the amount of added Ag precursor, e.g., Silver nitrate (AgNO_3_), as shown in Figure S4 (Supporting Information). When the Cu:Ag molar ratio increases from 0.15 to 0.5, the corresponding Ag shell thickness increases from 5 to 15 nm. Moreover, the stable Cu–Ag NWs have been previously dispersed in an organic ink solution that was used to cast transparent, conductive films.^[^ ^45^ ^]^ The same principles can be utilized for fabricating e‐textiles.

**Figure 2:**
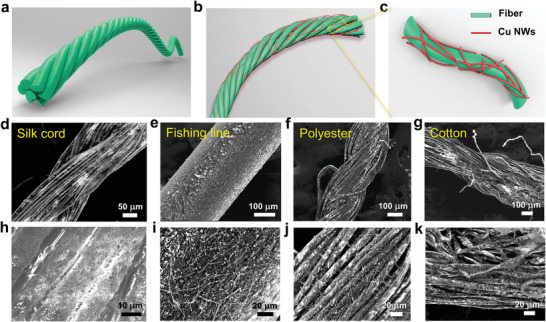
Illustration of thread a) and thread wrapped with Cu–Ag nanonets b,c). d–g) are low magnification SEM images of silk cord, fishing line, polyester thread, and cotton yarn in wrapping with Cu–Ag nanonets, respectively; h–k) are the corresponding high magnification SEM images of d–g), respectively.

A volatile solution was prepared as an ink for dispersing Cu–Ag NWs by mixing, e.g., acetone, ethanol, toluene, and nitrocellulose. The Cu–Ag NWs could be well dispersed in the ink after sonication and stably kept. The use of nitrocellulose could enhance the formation of Cu–Ag nanonets after the evaporation of the aqueous solvents. Threads were dipped into the Cu–Ag NWs ink solution for the wrapping of nanonets and lifted for drying, as shown in Figure 1d. Each dip‐wrapping process involves four essential steps (immersion, dwelling, withdrawal, and drying). It is found that the most critical two steps for achieving the best wrapping of Cu–Ag nanonets on the thread fibers are the withdrawal and drying. During withdrawal stage, it includes the critical balance of draining forces and entraining forces. Draining forces aim to draw the ink away from the threads and back toward the solution owing to gravity. Conversely, entraining forces are those that aim to retain conductive ink on the fibers owing to the surface tension of solution. The final coated thickness *D* of each dip‐wrapping depends on the velocity of withdrawal *V*, solution coefficient *k*, and the evaporation rate of the solvent *E*, which can be given by^[^ ^46^ ^]^

(1) $$D=k\frac{E}{LV}$$

(2) $$k=\frac{cM}{\alpha \rho }$$

where *L* is the width of the coated layer. *α*, *ρ*, *c*, and *M* are the porosity of the deposited layer, density of solute (Cu–Ag NWs and nitrocellulose), total concentration of solute and molar weight of the solute, respectively. Hereby, the concentration of nitrocellulose ink is the critical factor to blend homogeneous Cu–Ag nanonets, which then could tightly attach onto the surface of fibers and form conductive network, as shown in **Figure** **2**b,c. For our case, the optimal nitrocellulose concentration has been determined to be about 4.87 g L^−1^. Based on this fixed concentration, the coated thickness of Cu–Ag nanonets could be accurately controlled by adjusting the withdrawal velocity of thread away from the ink surface, which shows the method of fabricating conductive textiles has excellent reproducibility. During the drying process outside the surface, nitrocellulose forms an ultrathin layer and provides the capillary forces,^[^ ^40^, ^47^ ^]^ which enhances the tight binding of the interlaced Cu–Ag nanonets. This simple dip‐wrapping process effectively drives the thread conductive and the conductivity is proportional to the wrapping number (Figure 1f), which could meet different industrial demands. The resistance of the thread can become lower than 200 ohm cm^−1^ after just 5 times wrapping. Meanwhile, due to the high transparency of Cu–Ag nanonets, the thread could well preserve the original color. However, as the wrapping time becomes large, the color turns light red (Figure 1e). As presented in Figure 1g; and Figures S5 and S10 (Supporting Information), the light‐emitting diode (LED) indicators are successfully lit with the conductive threads in different colors, indicating the excellent conductivity and vivid color of threads wrapped with Cu–Ag nanonets.

In order to prove the universality of this method, various types of threads, including silk cord, fishing line, polyester thread, and cotton yarn, were modified with Cu–Ag nanonet wrapping and their surface morphologies were compared. Figure 1h; and Figures S5 and S14 (Supporting Information) show photographs of different types of conductive threads after treatment. One can see that their original characteristics such as color, gloss as well as softness have been well retained. Figure 1i summarizes the variation of the electrical performance of corresponding conductive threads as a function of number of coatings. As the number of coatings increases, the resistance of all threads decreases gradually. It is found that at the first three dip‐wrapping cycles, the resistance decreases much more steeply and after the third cycle, the resistance decreases steadily following a horizontal asymptote relationship. After 20 times wrapping, various types of conductive threads have similar conductivity (Figure S7, Supporting Information). Especially, the silk cord possesses the best conductivity in the lowest resistance over all threads, which is closely attributed to the high quality of its natural fibers (Figure 2d,h). The change in quality as a function of coating number was obtained for various threads, as shown in Figure S9 (Supporting Information). With the increase of coating numbers, the quality of the conductive thread increases slightly following a linear relationship. While, the threads still preserve the lightweight.

Figure 2a–c illustrates the structure of a thread and the geometry in wrapping with Cu–Ag nanonets. A thread is consisted of a bunch of twisted fibers and the nanonets can be wrapped on the outmost surface of the entire thread and the sidewall surface of each fiber. Figure 2d–g shows the SEM images of the silk cord, fishing line, polyester thread, and cotton yarn in wrapping with Cu–Ag nanonets, respectively. One can see that Cu–Ag nanonet could form uniformly on the different types of threads, leading to conductivity. By comparison, we see that among all types of threads, the fibers in the silk cord have the smoother and more compact surface. Whereas the fibers in the polyester thread and cotton yarn appear rather short and hairy here and there. Due to these smooth long fibers and shiny surface, the conductive silk cord could achieve the lowest resistance. Since the cotton yarn has a large number of short fiber tails stretching out, the wrapping of nanonets on these parts do not contribute much to the conductance and consequently, leading to relatively lower conductivity. The zoom‐in SEM images (Figure 2h–k; and Figure S15, Supporting Information) reveal the microscopic morphology of fibers with Cu–Ag nanonets. It is happy to find that the Cu–Ag nanonets could smoothly, uniformly and tightly coat not only on the outer surface of the thread but also on all the surfaces of every fiber like net tights owing to wettability of silk cord and capillary forces from ink solution. This clearly confirms the structure of the fiber wrapping with Cu–Ag nanonets as shown in Figure 2c. It is benefit from the solution coating method which enables the Cu–Ag NWs solution to easily go through the spaces between the twisted fibers and arrive at the hidden surfaces inside the thread.

**Figure 3:**
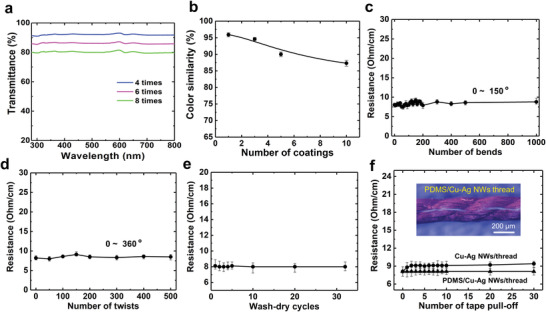
a) Transmittance spectra (280–800 nm) of fishing line coated by Cu–Ag nanonet with different coating numbers. b) The color similarity of conductive silk threads as a function of the wrapping number of Cu–Ag NWs (*n* = 3). Flexibility tests with bending c) and twisting d), stability tests with washing‐drying in water e) and tape test f) for conductive silk cord, respectively (*n* = 3). The inset of f) is optical microscope (OM) image of conductive thread coated with PDMS/Cu–Ag NWs.

The colorful thread is related to the high transparency of Cu–Ag nanonets. On account of the inconvenience in measuring the transmittance of a single conductive thread, a slit method was developed for determining the transmittance of the nanonets on a thread, as shown in Figure S16 (Supporting Information). A short slit in a width consistent with that of a thread was opened on a black plate. Fishing line was employed as the substrate for Cu–Ag nanonet wrapping and optical tests due to its high transparency and smooth surface (Figure 2e). Conductive fishing lines with different coating cycles of Cu–Ag nanonets were prepared and fixed on the slit. In this way, the transmittance of Cu–Ag nanonet layer on a thread could be obtained, as presented in **Figure** **3**a. One can see that the transmittance is as high as over 91% in 4 coating cycles and the transmittance decreases slightly with the increase of coating times. Even after wrapping for 8 cycles, the transmittance could still retain 80% (with a resistance of ≈70 ohm cm^−1^) in the visible band. The color similarity of conductive silk cord with different wrapping numbers were measured by commercial color sensor, as shown in Figure 3b; and Figure S17 (Supporting Information). One can see that the color similarity slightly decreases with the increasing wrapping times. Even though, the color similarity can still retain 87% after 10 times wrapping, which indicates the excellent preservation of original color of threads. These results confirm that the high transparency of Cu–Ag nanonets is the main reason promising the preservation of the original color and gloss of the wrapped threads.

Mechanical stability, flexibility, and adhesiveness were further tested. Figure 3c,d shows the change of resistance for the conductive silk cord under repeated bending (angle of 150°) and twisting (angle of 360°). Both bending and twisting tests will demonstrate the robust stability and flexibility of Cu–Ag nanonets with unchanged resistance, even after 1000‐time bends or 500‐time twists. Generally, textiles in production and clothes in daily use always require washing and drying. In order to further test the durability under washing, a stir‐washing test was setup for simulating the processes of stirring, washing, and drying in a washing machine (Figure 3e; and Figure S18, Supporting Information). After 30 cycles of washing and drying, it can be confirmed that stable conductivity of silk cord reflects the excellent adhesion of Cu–Ag nanonets on the surface as well as the excellent hydrophobicity of cellulose. The adhesion property was then further tested by peeling with Scotch tape (Figure 3f). The resistance of conductive threads only increases by 1 ohm in the first 3 times of the tape off‐peeling and then becomes stable. This means that at the beginning, those weakly adhered NWs and those NWs on the out‐stretching fibers are removed, which slightly deteriorates the electrical property. In order to further improve the adhesion of the nanonets, ultrathin PDMS layer^[^ ^48^, ^49^ ^]^ was coated on the outmost surface of thread and on Cu–Ag nanonets. The PDMS layer has very high transmittance and could be uniformly coated on the surface. As a result (triangle line in Figure 3f), conductive silk cords with PDMS coating layer exhibit rather stable conductivity under peeling test. The durability of these conductive threads has been good enough for further e‐textile applications.

**Figure 4:**
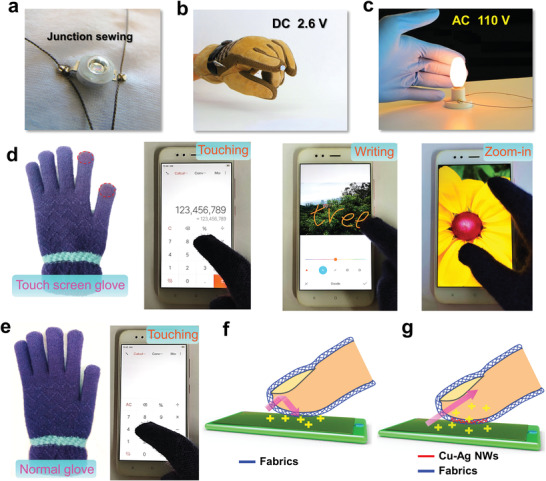
The optical images of conductive threads used in lighting LED on wearable clothes a), glove b), and lighting a small bulb electrical circuit c). d) Photographs of touch screen glove made by Cu–Ag nanonets, operating smart phone, e.g., touching, writing, and zoom‐in, respectively (from left to right). e) Photographs of normal glove (left) and operating smartphone invalidly (right). Schematic working mechanism of normal glove f) and touch screen glove g), respectively.

Such a soft and flexible conductive thread could be used as circuit components in wearable electronic fabrics by traditional tailoring techniques such as sewing and weaving. As shown in **Figure** **4**a,b, conductive threads are used to sew a LED on the clothes or a glove without any tin soldering, which can light up the white or blue LEDs with a direct current (DC) voltage of 2.6 V. Beyond the DC circuit, the conductive thread can even be used to connect a light bulb by screw and the alternating current (AC) voltage of 110 V is applied stably for lighting (Figure 4c).

Regarding gloves, we all have the experience that in the cold winter, fingers in a glove have a problem in operating the touch screen of intelligent devices such as smartphone (Figure 4e; and Video S1, Supporting Information). This is mainly due to nonconductivity of the glove fabrics (Figure 4f). As we know, the dominant control technique of touch screen requires the conductivity of the finger to process a command.^[^ ^50^, ^51^ ^]^ Therefore, a touch‐screen glove should be conductive in the part of the finger tips. Here, we designed a touch‐screen glove by activating the conductivity of tip areas on the thumb and index fingers of normal glove made of polyester textile via the drop‐wrapping Cu–Ag nanonet ink (Figure 4d). As shown in Figure 4g, the conductive textile will conduct the charges between screen and finger tip, which leads to capacitance response on the touch screen. As a result, one can operate the smartphone fluently with this touch‐screen glove, e.g., touching scrolling, writing, or two‐finger zooming gestures (Figure 4d; and Videos S2 and S3, Supporting Information). The low temperature stability was tested by operating the smartphone with this glove in a freezer under −20 °C. This glove works well for more than 1 h and over 1000 times of various gestures, indicating the excellent frost‐resistance and wear‐resistance properties of Cu–Ag nanonets wrapped textiles.

**Figure 5:**
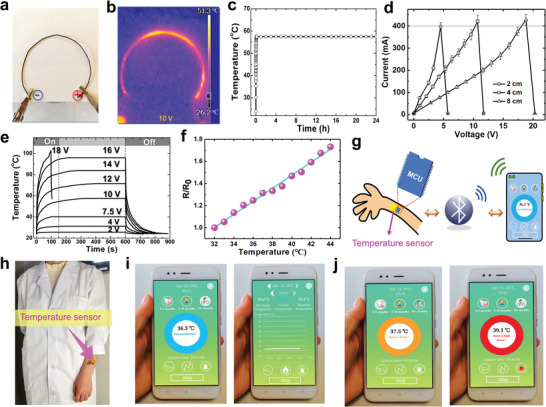
Photograph a) and IR thermal image b) of ring heater made by silk cord with Cu–Ag nanonet wrapping. c) Variation of temperature for heating ring with different durations at 10 V (*n* = 4). d) Current as a function of cord length under different Voltage (*n* = 4). e) Time‐dependent temperature profiles of the conductive silk cord coated Cu–Ag nanonets operated by different voltages (*n* = 4). f) Relative resistance variation of temperature sensor under different temperature (*n* = 3). g) Schematic illustration of the operation process of the temperature sensor. h) Temperature sensor is placed on the arm. i,j) are the real‐time smartphone user interfaces when different body temperatures are monitored.

The colorful conductive threads have potential for the application as wearable heaters owing to the good electrical performance and biocompatibility.^[^ ^52^, ^53^, ^54^ ^]^ As for nanowires, it has been further found that the lower rate of ion production compared to that of nanoparticles will have even weaker cytotoxicity to living cells.^[^ ^55^, ^56^ ^]^ A linear heating thread was prepared and the DC voltage was applied to control the temperature (**Figure** **5**a). The infrared radiation (IR) thermal image in Figure 5b shows the temperature distribution of the ring heater under 10 V. It is clearly seen that the heating temperature can reach 50 °C quickly and is relatively uniform. After working for 24 h, the ring heater still shows a good stability (Figure 5c). The heating current as a function of cord length was recorded under different external voltages, as shown in Figure 5d. We find that as the cord length increases, the current increasing rate slows down, which is inversely proportional to the line resistance. The conductive silk cords with different lengths have nearly the same maximum current (400 mA), which is the failure point of conductive threads. This indicates that the extremely high heat will damage the Cu–Ag nanonets and break the conducting network.

Usually, temperature of the heater can be determined by two processes: inflow and storage of the electrical energy, and outflow and releasing of the energy through heat transfer to air.^[^ ^57^ ^]^ As shown in Figure 5e, when applying different voltage, the temperature of single conductive silk cord will rise until the heating system is balanced between input power (Joule heat of the Cu–Ag nanonets) and heat loss.^[^ ^58^, ^59^ ^]^ The balanced temperature can be defined as the saturation temperature^[^ ^60^ ^]^

(3) $$T_{\mathrm{sat}}-T_{0}=VI/2\pi rlh$$

where *T*_sat_ is the saturation temperature, *T*_0_ is the initial temperature (room temperature), *V* is the applied voltage, *I* is the corresponding current, *h* is the convective heat transfer coefficient, *r* and *l* are the radius and length of conductive thread, respectively. But when the actual temperature exceeds *T*_sat_, the extra heat cannot be dissipated rapidly. Transient heat congestion will lead to the local fusing of metal nanonets. Here, when the applied voltage is over 18 V, the temperature will exceed 100 °C. At this point, the Cu–Ag nanonet will be broken and the heater fails. On the other hand, it is noted that the saturation temperature can be adjusted easily by applied voltage to meet different temperature requirements. The heaters fabricated by conductive threads have an excellent heating performance and high stability, which can be used in industrial miniature heating devices.

We further observed that the conductive thread is not only able to give out heat but also electrically sensitive to temperature. This feature makes it very suitable for fabricating temperature sensors, especially for the body temperature monitoring. Available traditional thermometers could be inaccurate with the error of ±0.3~ ±1.0 °C (infrared forehead thermometer and temperature indicating strip), unsafe (mercury thermometer), or slow response with time about 3–15 s (digital thermometer and ear thermometer). To evaluate the thermal sensitivity of our conductive thread and textile, a square piece of conductive cotton cloth (2 × 2 cm^2^) was prepared by the dip‐wrapping process and the relative resistance variation as a function of temperature was measured, as shown in Figure 5f. It shows a clear linear relationship and could be used to fit the temperature coefficient of resistance (*TCR*)^[^ ^61^ ^]^ by

(4) $$TCR=\frac{R-R_{0}}{R_{0}\cdot \left(T-T_{0}\right)}$$

where *R* is the real‐time resistance, *R*_0_ is the initial resistance, *T* and *T*_0_ are the real‐time temperature and the initial temperature, respectively. The *TCR* of this conductive fabric is about 0.0589 °C^−1^, which is much high than nanostructured conductors (Table S2, Supporting Information). It is found that this temperature sensitive region covers the human body and common body temperature range, so that a wearable real‐time thermometer was fabricated based on conductive polyester textile for remote monitoring of body temperature, as shown in Figure 5g. The sensing textile was integrated with a microcontroller unit (MCU chip) and a Bluetooth communicator. Hence, the skin thermometer could be directly attached on the skin by medical paste (Figure 5h; and Figure S20, Supporting Information) or sewn on the inner side of the sleeve. A smartphone app was designed to remotely control and display the real‐time body temperature on a friendly interface through Bluetooth communication module (Figure 5i,j). By this means, the body temperature variation could be shown in real‐time curve and symptom warning signals will appear when the temperature is abnormal. Blue, orange, and red colors represent “Normothermia” (< 37.5 °C), “Fever” (≈37.5–38.5 °C) and “Hyperthermia” (> 38.5 °C), respectively. We used a hair drier to heat the arm skin for mimicking the fever states. The smart thermometer demonstrates an accurate temperature sensing and correct immediate warning colors, as displayed in Figure 5j; and Video S4 (Supporting Information). Compared with the test results of mercury thermometer, the body temperature tested by temperature sensing system have an error of ±0.3 °C. This product could make the body temperature monitoring more convenient and precise, especially for the effective fever screening in public places during this COVID‐19 pandemic.

**Figure 6:**
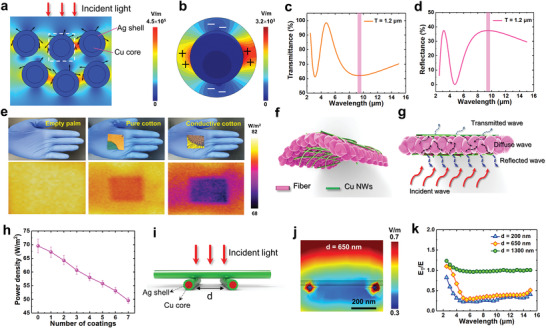
a) Electric field distribution of Cu–Ag nanonets with 2D model. The arrows indicate reflected path of incident light. b) Amplification of white dotted line in a). Transmittance c) and reflectance d) of Cu–Ag nanonets with thickness of 1.2 µm under mid‐infrared. e) Photographs (up) and IR thermal images (down) of empty plan, pure cotton fabric, and cotton fabric coated with Cu–Ag nanonets, respectively. f) Schematic of conductive fabrics coated with Cu–Ag nanonets. g) Schematic of IR reflected on conductive fabrics. h) Power density as a function of wrapping number of Cu–Ag nanonets (*n* = 4). i) Model structure of crossed Cu–Ag nanonet. j) Transverse electric field distribution of Cu–Ag nanonet in mid‐infrared. k) Relative transmission changes of Cu–Ag nanonet with different distance of NWs in mid‐infrared.

Clothing plays the important role in keeping warm of human body by preventing heat exchanging between body and environment, which generally depends on the adiabaticity and thickness of textiles. It has been learned that more than 50% of the heat generated by human body is dissipated through infrared radiation,^[^ ^62^, ^63^ ^]^ mainly from mid‐infrared wavelength of 9.6 µm. A 2D model of Cu–Ag nanonet was constructed (Figure S22, Supporting Information) and the electric field distribution under mid‐IR was calculated by finite element method, as shown in **Figure** **6**a,b. One can see that a part of incident wave is reflected by Cu–Ag nanonets due to high reflectivity of silver shell, as illustrated by the arrows. The reflection includes direct reflection and scattered reflection (the light propagation is changed toward the lateral direction). Most interestingly, it is found that a layer of strong electric field forms between lateral Cu–Ag NWs nanonet, indicating a significant light trapping and absorbing within the nanonet. Consequently, the transmitted light below the nanonet becomes very few. By looking into the detailed electric field distribution of a single Cu–Ag NW (Figure 6b), we observe a synergistic effect of local surface plasmon on the Ag shell layer, which exhibits a typical dipole resonance along *x* axis. This local surface plasmonic effect in the Cu–Ag core–shell structure provides strong electromagnetic resonant absorption of mid‐IR waves. The transmittance, reflectance, and absorbance of the Cu–Ag nanonets in different thickness were calculated (Figure S23, Supporting Information). It can be seen that continuous shift of dip in the spectrum and the increase of the number of resonant nodes takes place with the increasing nanonet thickness, showing the tunability of the transmitting window.^[^ ^64^, ^65^ ^]^ From Figure 6c,d, it is determined that with a thickness of about 1.2 µm, a band in high reflectivity can well cover the wavelength of 9.6 µm, which is beneficial for reducing IR transmission. Based on the above understanding, we fabricated a novel warm fabric wrapped with Cu–Ag nanonets (Figure 6f). And the fabrication method of the warm fabric is exactly the same as previous mentioned, by dip‐withdraw coating of cotton fabric with Cu–Ag nanonets. It not only possesses high air permeability, thin thickness, and biocompatibility, but also can reduce heat loss by reflecting infrared radiation from human body.

The fabric was placed on the palm and the screening effect on infrared radiation was detected by using an IR camera, as shown in Figure 6e. It is determined that the infrared thermal radiation from the palm is in a power density of 81 W m^−2^. Compared with common cotton cloth, the transmitted IR through the cotton textile with Cu–Ag nanonets has been reduced to 69 W m^−2^, indicating the effective blocking of thermal dissipation. Incident IR wave is partially reflected by the surface of textiles due to high reflectivity of Ag shell layer (Figure 6g). Meanwhile, another part of scatter reflective IR goes within the textiles and is absorbed after multiple reflection and absorption. The energy of thermal radiation is then converted back into heat energy, forming a warm fabric itself. To explore the influence of thickness and spacing of Cu–Ag nanonets on thermal isolation effect, the conductive fabrics were suspended on a heating plate (40 °C) and the power density of conductive fabrics was measured. From Figure 6h; and Figure S24 (Supporting Information), one can see that the power density of conductive fabrics decreases lineally with increasing coating number of Cu–Ag nanonets, indicating the improvement of thermal blocking. Besides, from Table S3 (Supporting Information), it can be found that our conductive textiles have wider temperature regulation range and better performance about keeping warm of human body. We found that the distance between NWs will also gradually decrease at the same time. Electric field simulations were performed to investigate the crossed Cu–Ag nanonets in various densities (Figure 6i; and Figure S25, Supporting Information). The diameter of Cu core was set to 40 nm and the Ag shell thickness is 5 nm. Figure 6j; and Figure S26 (Supporting Information) shows the cross‐sectional images of the electric field distribution on the Cu–Ag nanonet under downward incidence of 9.6 µm wave. One can see that when the distance (*d*) between the Cu–Ag NWs becomes narrower than 650 nm, the transmission electric field intensity below the Cu–Ag nanonet appears very weak. More than half of the electric energy has been largely blocked. Figure 6k shows the plots of the relative transmission (*E*_T_/*E*) in the mid‐infrared band as a function of NWs distance. It is found that for *d* < 650 nm, more than 75% IR energy (over 5–10 µm) could be well filtered. Besides, when the distance becomes larger than 1300 nm, total transmission including slight resonance enhancement will occur at wavelength from 4 to 15 µm. Models of pure Cu NWs and PDMS NWs (Figure S27, Supporting Information) were calculated for comparison, which revealed that the Ag shell layer plays the critical role in reflecting IR light. Owing to the excellent IR reflectance of Cu–Ag nanonets, infrared invisibility clothes could be achieved by using these conductive textiles.

## 3 Conclusion

In conclusion, Cu–Ag NWs were used to develop a fast, room‐temperature method to obtain colorful conductive threads and fabrics for wearable electronics. Due to the stable Ag shell surrounding the Cu NWs, excellent conductivity was achieved without the need for post treatment steps. Fibers of thread were tightly wrapped with Cu–Ag nanonets through a dip‐wrapping process in a volatile solution. The obtained conductive threads not only retained the original features (e.g., color, gloss, softness) of the textiles, but also enhanced their mechanical stability, dry‐wash durability, and sewability. Furthermore, e‐textile devices such as a fabric heater, touch screen gloves, a wearable real‐time temperature sensor, and warm fabrics were fabricated with excellent properties. These colorful conductive threads and fabrics wrapped with Cu–Ag nanonets are a promising candidate for future wearable electronics.

## 4 Experimental Section

### Chemicals

Copper(II) nitrate hemipentahydrate (Cu(NO_3_)_2_ · 2.5 H_2_O, ≥98.0%), ethylenediamine (EDA, ≥99.5%), hydrazine (35 wt% in H_2_O), polyvinylpyrrolidone (PVP, avg MW 10 000), silver nitrate (AgNO_3_, ≥99.0%), L‐ascorbic acid (99.0%), ethyl acetate, pentyl acetate, and nitrocellulose (NC) were all obtained from Sigma‐Aldrich. Sodium hydroxide (NaOH, 99.0%) was purchased from NOAH Technologies Corporation. *N*,*N*‐Diethylhydroxylamine (DEHA, >95.0%) was obtained from TCI America. Isopropyl alcohol (IPA, ACS reagent), acetone, ethanol, and toluene were purchased from BDH VWR International.

### Synthesis of Cu–Ag NWs

Cu NWs were synthesized using a scaled‐down version of a solution‐based method.^[^ ^43^ ^]^ In summary, solutions containing 0.5 mmol Cu(NO_3_)_2_, 0.6 mol NaOH, and 4.16 mmol glucose were prepared in 5, 75, and 7.5 mL of deionized water, respectively. EDA (0.75 mL), the sodium hydroxide solution, and the glucose solution were sequentially added to Cu(NO_3_)_2_ solution and vortexed well after each addition. The mixture was immediately placed in a 60 °C oven for 1 h. After cooling to room temperature, the Cu NWs were rinsed three times through centrifugation with an aqueous solution containing 3 wt% PVP and 1 wt% DEHA, discarding the supernatant each time. Finally, the Cu NWs with diameter of 40 nm and average length of 20 µm were suspended in the PVP/DEHA solution at a concentration of 0.8 mg mL^−1^.

A thin Ag coating was then applied to the Cu NW surfaces through an electroless deposition method.^[^ ^44^ ^]^ To achieve the Ag coating, 5.5 mL of 1 m L‐ascorbic acid and 2 mL of 5 wt% PVP solution were added to 2 mL of 0.8 mg mL^−1^ Cu NW solution and stirred for 3 min. After that, 0.5 mL of 25 mm AgNO_3_ solution was added into the mixture and stirred for 3 min. Then, the Cu–Ag NWs with diameter of Cu core of 40 nm, Ag shell thickness of 5 nm and average length of 20 µm were obtained. The Cu–Ag NWs solution were vortexed to remove aggregation and the resulting Cu–Ag NWs were resuspended in a volatile ink containing acetone, ethanol, toluene, ethyl acetate, pentyl acetate, IPA, and NC at a concentration of 8 mg mL^−1^.

### Preparation of Conductive Threads

The conductive threads can be obtained by dipping into Cu–Ag NWs ink solution. First, the thread was fixed on one end of the mechanical arm that can move vertically up and down in a constant speed. Then the thread was immersed into Cu–Ag NWs ink solution at a speed of 2 cm s^−1^ and kept in it for 5 s. Finally, the thread was withdrawn from the Cu–Ag NWs ink solution at a speed of 2 cm s^−1^ and then exposed in the air for drying for 1 min. All the original threads in the experiment are only washed by alcohol and deionized water, respectively, and without other surface modification.

### Preparation of Touch‐Screen Glove

The touch‐screen glove was fabricated by modifying the normal glove. The conductivity of tip areas on the thumb and index fingers of normal polyester glove was activated via the drop‐wrapping Cu–Ag nanonet ink. After the ink evaporated at room temperature, touch‐screen glove was obtained.

### Preparation of Temperature Sensing Cloth

The conductive fabrics were prepared by dip‐wrapping process. The cotton fabrics (2 × 2 cm^2^) were dipped in the Cu–Ag NWs ink followed drying. The conductivity of fabrics could be tailored by adjusting the number of dip‐coatings. Then the copper tape was put on the both ends of conductive fabrics as electrodes. The whole temperature sensing system includes temperature sensor (conductive fabrics), test module, microcomputer module, Bluetooth module, and display module.

### Washing‐Drying Test

In order to test the durability under washing, a stir‐washing test was setup for simulating the process of stirring in a washing machine, including washing and drying. As shown in Figure S18 (Supporting Information), conductive silk cord was put into a container with tap‐water and detergent, and then put them on the magnetic stirrer with 500 rpm at room temperature. After 10 min, conductive thread was taken out and placed on a hot plate with 50 °C for drying for 30 min. Then the process of stirring, washing, and drying were repeated.

### Characterization

The morphology and microstructure of the Cu NWs and Cu–Ag NWs were measured by field emission SEM (Hitachi S‐4800). The Ag shell thickness of Cu–Ag NWs was investigated by TEM (TECNAI F‐30). The ultraviolet visible (UV–vis) spectrophotometer (Varian Cary) was used for transmittance of conductive threads. The temperature of samples was obtained by IR thermal imager (Fluke Ti90). The Keithley 2450 system was applied to provide the voltage source and measure the resistance as well as current. The color similarity of samples were measured by commercial color sensor (KEYENCE LR‐WF10).

### Statistical Analysis

Statistical analysis was compiled on the means of the data obtained from at least three independent experiments using Origin software. All values were expressed as the mean ± standard deviation (SD) of individual sample. The sample size (*n*) numbers for each experiment were indicated in the figure legends.

### Study Participation

Prior to participation in the experiments, informed consent was obtained from the volunteer in all experiments.

## Conflict of Interest

The authors declare no conflict of interest.

## Supporting information

Supporting Information

Supplemental Video 1

Supplemental Video 2

Supplemental Video 3

Supplemental Video 4

## Data Availability

The data that support the findings of this study are available in the Supporting Information of this article.
