# Supplementary material for: Colorful Conductive Threads for Wearable Electronics: Transparent Cu–Ag Nanonets
Source: Adv Sci (Weinh). 2022 Jul 15;9(24):2201111. doi: 10.1002/advs.202201111 (PMC9405525; doi:10.1002/advs.202201111)
Supplement: Supplementary file 1 — Supporting Information [file ADVS-9-2201111-s004.pdf]

ADVANCED SCIENCE
Open Access

Supporting Information

for *Adv. Sci.*, DOI 10.1002/advs.202201111

Colorful Conductive Threads for Wearable Electronics: Transparent Cu–Ag Nanonets

Yan Tang, Bin Guo, Mutya A. Cruz, Han Chen, Qicheng Zhou, Zefeng Lin, Fuchun Xu, Feiya Xu, Xiaohong Chen, Duanjun Cai*, Benjamin J. Wiley* and Junyong Kang*

Supporting Information

Colorful Conductive Threads for Wearable Electronics: Transparent Cu-Ag Nanonets

Yan Tang, Bin Guo, Mutya A. Cruz, Han Chen, Qicheng Zhou, Zefeng Lin, Fuchun Xu, Feiya Xu, Xiaohong Chen, Duanjun Cai, Benjamin J. Wiley, and Junyong Kang*

Y. Tang, B. Guo, H. Chen, Q. C. Zhou, Z. F. Lin, Prof. F. C. Xu, F. Y. Xu, Prof. X. H. Chen, Prof. D. J. Cai, Prof. J. Y. Kang
Fujian Key Laboratory of Semiconductor Materials and Applications, CI center for OSED, College of Physical Science and Technology, Xiamen University, Xiamen 361005, China.
E-mail: dcai@xmu.edu.cn

Dr. M. A. Cruz, Prof. B. J. Wiley
Department of Chemistry, Duke University, Durham, NC, 27708-0354, USA.

This PED file includes:

Figure S1. Schematic illustration of (a) Cu-Ag NWs, (b) cross section of Cu-Ag NWs.

Figure S2. (a) EDS spectrum of Cu-Ag NWs. (b) TEM images of Cu-Ag NWs.

Figure S3. FTIR spectra of cotton thread and conductive cotton thread coated with Cu-Ag NWs.

Figure S4. Plot of Ag:Cu molar ration versus Ag shell thickness (n = 20).

Figure S5. Photographs of different types of conductive threads obtained by different wrapping number (0, 1, 5, 12) of Cu-Ag nanonets.

Figure S6. Optical images of dispersion solution (a) and corresponding transparent conductive electrodes (b) of bare Cu NWs, Cu NWs with different Ag shell thickness (5 nm, 15 nm, 30 nm) and pure Ag NWs, respectively.

Figure S7. (a) Resistance as a function of wrapping number of Cu-Ag nanonets for different types of threads. (b) Magnified curves of red dotted line in (a).

Figure S8. (a) and (c) are the photographs of original silk threads produced by different weaving methods. The resistance at different positions of conductive silk thread 1 with different lengths (b) conductive silk threads 2 with different lengths (d), respectively.

Figure S9. Quality changes of conductive threads with coating numbers.

Figure S10. (a-b) Photographs of large-scale conductive thread. (c) Large-scale conductive thread used in electrical circuit.

Figure S11. Conductive fabrics used in electrical circuit.

Figure S12. Optical microscope (OM) image of conductive thread.

Figure S13. OM image of conductive fabric.

Figure S14. Photographs of different types of curved conductive threads coated by Cu-Ag nanonets with wrapping number of 12.

Figure S15. SEM image of the unraveled silk cord fibers coated with Cu-Ag nanonets.

Figure S16. Measuring device and method of transmittance of threads.

Figure S17. Photos of color similarity tests of conductive silk threads by commercial color sensor.

Figure S18. Photograph of stir-washing test.

Figure S19. The relative resistance changes of conductive silk cord under room temperature for one month (a), a constant condition with 85 ℃ and 85% relative humidity for 72 h (b).

Figure S20. Photograph of temperature sensor on the arm.

Figure S21. The test results of body temperature on a volunteer by the temperature sensing system made by Cu-Ag nanonets (a) and a mercury thermometer (b) at the same time.

Figure S22. Simulation of Cu-Ag nanonets with 2-D model.

Figure S23. (a-d) Transmittance of Cu-Ag nanonets with thickness of 90 nm, 500 nm, 1.2 μm and 2 μm under mid-infrared wavelength, respectively. (e-h) Reflectance of Cu-Ag nanonets with thickness of 90 nm, 500 nm, 1.2 μm and 2 μm under mid-infrared wavelength, respectively. (i-l) Absorbance of Cu-Ag nanonets with thickness of 90 nm, 500 nm, 1.2 μm and 2 μm under mid-infrared wavelength, respectively.

Figure S24. IR thermal images of conductives fabircs with different wrapping number of Cu-Ag nanonets.

Figure S25. Model structure of crossed Cu-Ag nanonet.

Figure S26. Transverse electric field distribution of Cu-Ag nanonet with different distance of NWs in mid-infrared.

Figure S27. Relative transmission changes of Cu NWs network (a) and PDMS network (b) with distance of 650 nm in mid-infrared.

Video 1. Video of operating smartphone with normal glove.

Video 2. Video of operating smartphone with touch screen glove made by Cu-Ag nanonets.

Video 3. Video of operating smartphone with touch screen glove made by Cu-Ag nanonets.

Video 4. Video of testing body temperature with sensor fabricated by Cu-Ag nanonets.

Table S1 Summary of optoelectronic property of conductive textiles based on silver in recent years.

Table S2 Summary of temperature sensor based on conductive textiles in recent years.

Table S3 Summary of textiles with temperature regulating property in recent years.

References:

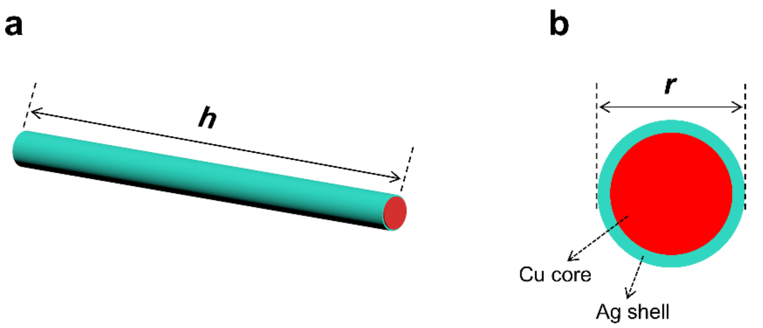

Figure S1. Schematic illustration of (a) Cu-Ag NWs, (b) cross section of Cu-Ag NWs.

As we know, copper ($5/kg) is 1000 times more abundant and 100 times less expensive than silver ($500/kg), while only 6% less conductive.[1-3] Take an example that the diameter of Cu-Ag NWs is 50 nm (the diameter of Cu core is 40 nm and Ag shell thickness is about 5 nm) and the average length of Cu-Ag NWs is 20 µm. We assume that the nanowire is a cylinder (**Figure S1**). The cost of each nanowire can be calculated by:

$$P = c * \pi r^2 * h * \rho \quad (1)$$

Where c is the cost of metal per kilogram, π is the constant (3.14), r and h are the radius and length of nanowire, respectively. ρ is the density of metal (Ag ~ 10.49 g/cm^3 and Cu ~ 8.96 g/cm^3). We find that the cost of pure Ag nanowire could be about 2.8 times higher than that of Cu-Ag nanowire.

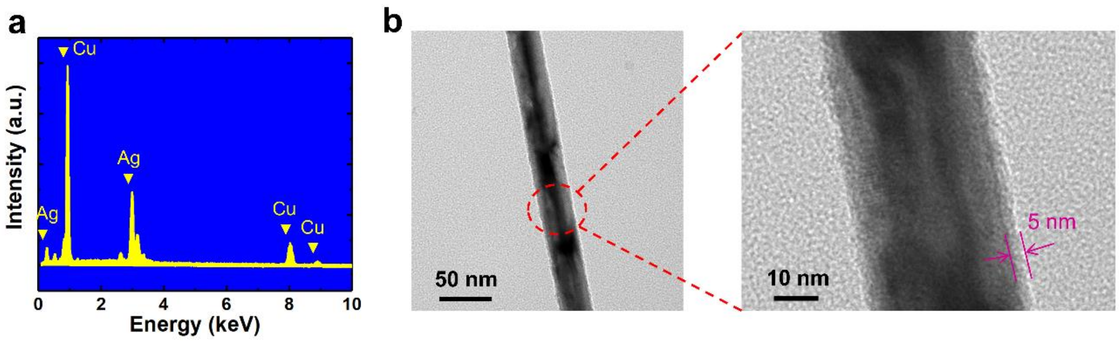

Figure S2. (a) EDS spectrum of Cu-Ag NWs. (b) TEM images of Cu-Ag NWs.

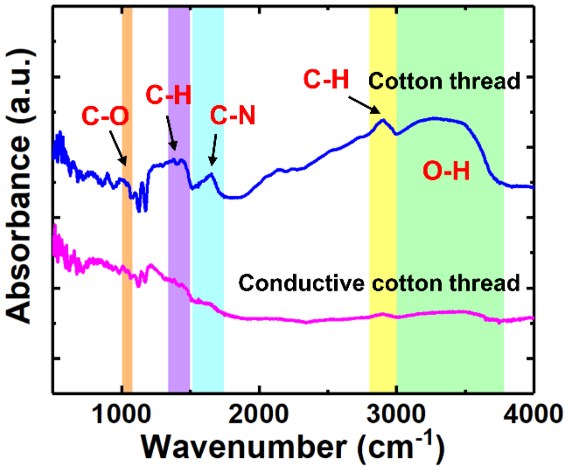

Figure S3. FTIR spectra of cotton thread and conductive cotton thread coated with Cu-Ag NWs.

FTIR measurements for cotton threads before and after Cu-Ag NWs wrapping, respectively, were measured to characterize the chemical bond information. As shown in **Figure S3**, the original chemical bonds in the cotton thread could be identified and the characteristic peaks of the functional groups appeared at around 1042, 1384, 1653, 2903 and 3000-3800 cm^{-1}, which could be assigned to the signals from C-O stretch, C-H bend, N-H bend, C-H stretch and overlapping of the O-H stretch and N-H stretch, respectively.[4, 5] After Cu-Ag NWs wrapping, we did not observe the formation of new chemical bonds between threads and Cu-Ag NWs. However, we can see that after Cu-Ag nanonet wrapping, all characteristic peaks of cotton thread have been greatly weakened. This could be attributed to the high reflection of infrared by Cu-Ag NWs.

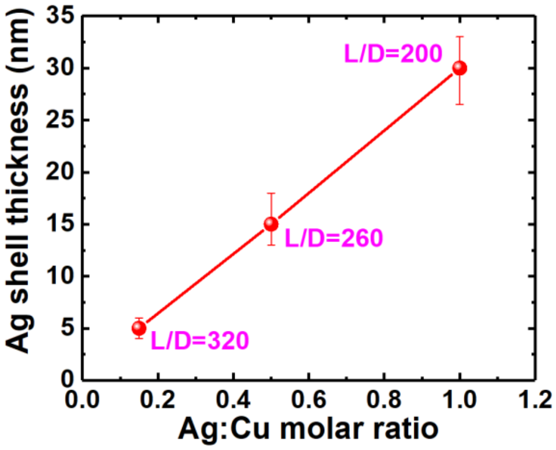

Figure S4. Plot of Ag:Cu molar ration versus Ag shell thickness (*n* = 20).

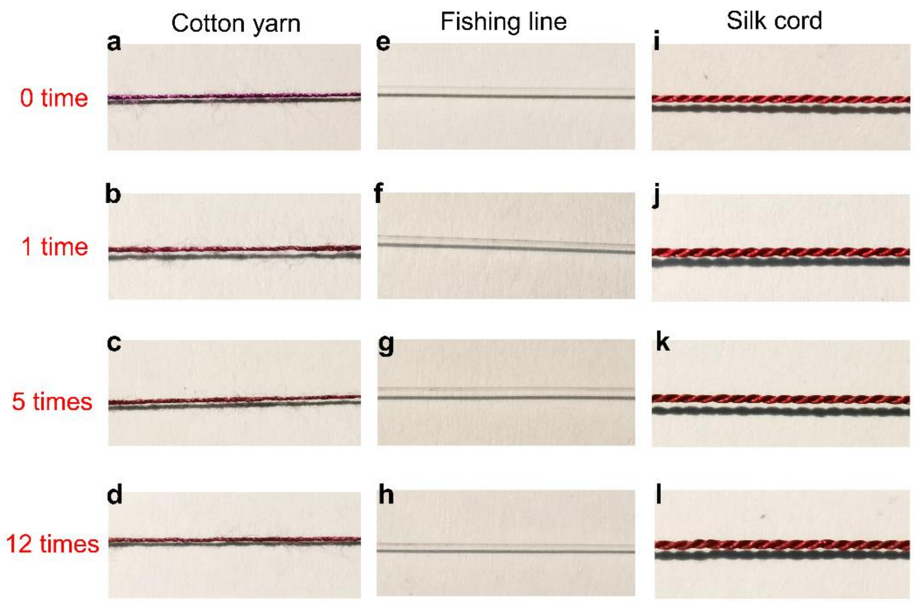

Figure S5. Photographs of different types of conductive threads obtained by different wrapping number (0, 1, 5, 12) of Cu-Ag nanonets.

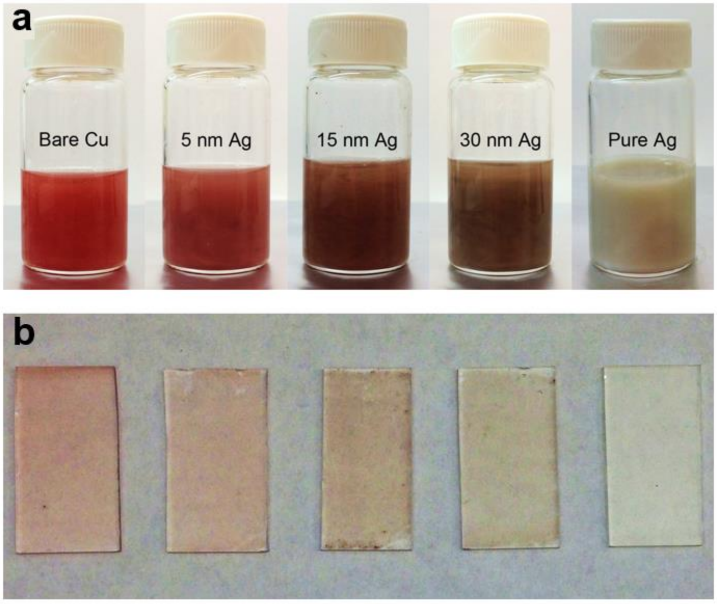

Figure S6. Optical images of dispersion solution (a) and corresponding transparent conductive electrodes (b) of bare Cu NWs, Cu NWs with different Ag shell thickness (5 nm, 15 nm, 30 nm) and pure Ag NWs, respectively.

The dispersion solution of pure Ag NWs is milk-white and the dispersion solution of bare Cu NWs is red. After coated with Ag layer, the color of Cu NWs will change.

And with the thickness of Ag shell layer increasing, the color of Cu-Ag NWs will change from red brown to grey brown gradually.

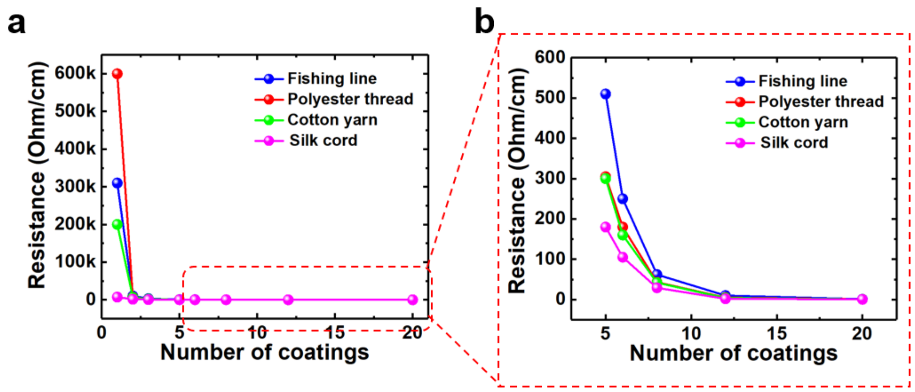

Figure S7. (a) Resistance as a function of wrapping number of Cu-Ag nanonets for different types of threads. (b) Magnified curves of red dotted line in (a).

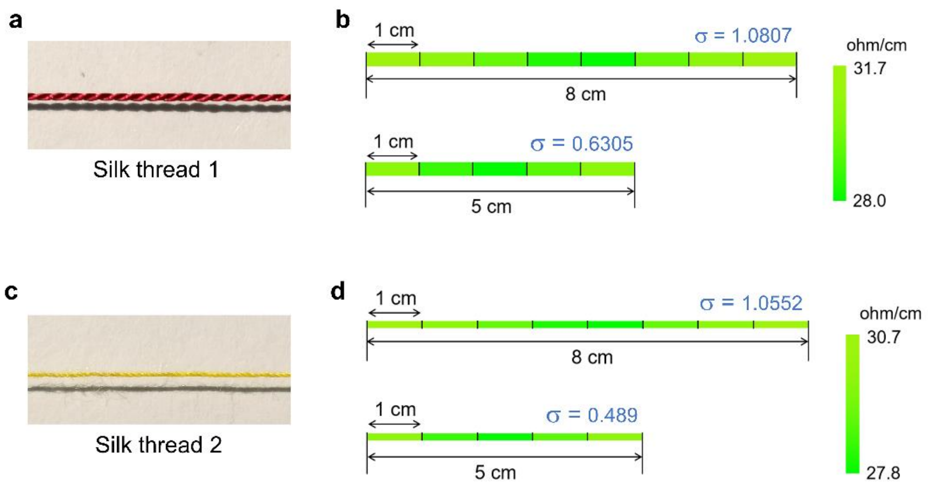

Figure S8. (a) and (c) are the photographs of original silk threads produced by different weaving methods. The resistance at different positions of conductive silk thread 1 with different lengths (b) conductive silk threads 2 with different lengths (d), respectively.

In order to prove the uniformity of conductivity of conductive thread, the resistances of different conductive silk threads and the same thread in various lengths were tested. The standard variance data of resistance of conductive threads was calculated, as presented in **Figure S8**. Here, silk thread 1 and silk thread 2 are produced by different weaving methods. According the formula of standard deviation (σ)

$$\sigma = \sqrt{\frac{1}{N}\sum_{i=1}^{N}(R_i - \bar{R})^2} \;, \tag{1}$$

$$\bar{R} = \frac{1}{N}\sum_{i=1}^{N} R_i \;. \tag{2}$$

where N is number of tested resistances, R is the resistance of conductive threads, and $\bar{R}$ is average resistance of conductive thread. Standard deviation of resistance based on conductive silk thread 1 with length of 8 cm and 4 cm are 1.0807 and 0.6305, respectively. Standard deviation of resistance based on conductive silk thread 2 with length of 8 cm and 4 cm are 1.0552 and 0.489, respectively. From the results, it shows that the resistance difference of conductive thread at different positions is very small and our conductive threads have a high uniformity of conductivity.

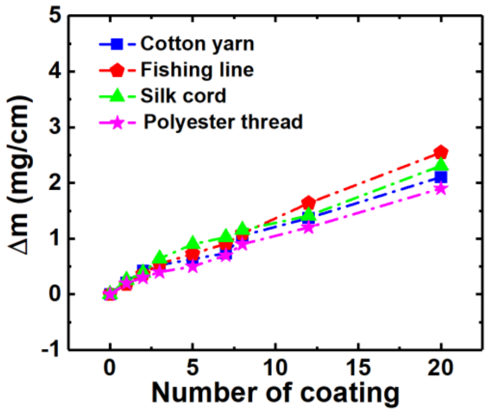

Figure S9. Quality changes of conductive threads with coating numbers.

The fishing line is the smoothest than other three types of threads (cotton, polyester and silk cord). However, on the other hand, its wettability and the bunch of fibers are much less than the other threads. During withdrawal stage, it includes the critical balance of draining forces and entraining forces. The entraining force for the fishing line is larger than draining forces because of the smooth surface. That is why the resistance of fishing line is larger. While, after several coating numbers, the conductivity of the fishing line becomes similar to those of the other threads.

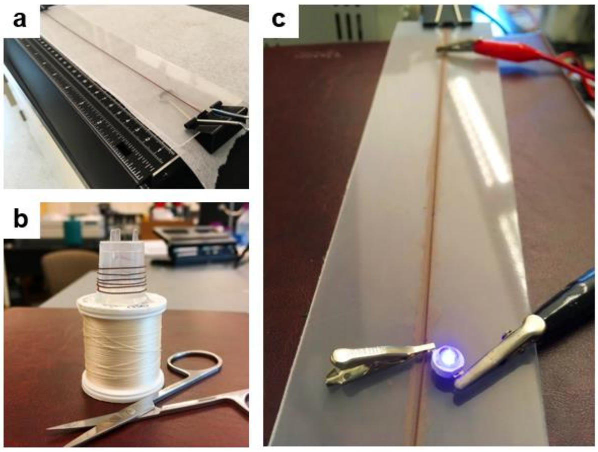

Figure S10. (a-b) Photographs of large-scale conductive thread. (c) Large-scale conductive thread used in electrical circuit.

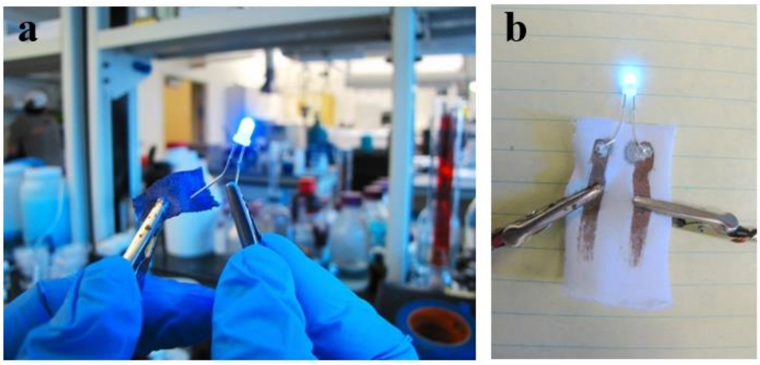

Figure S11. Conductive fabrics used in electrical circuit.

The same fabrication method by dip-coating can be utilized for fabricating large-scale conductive threads and conductive textiles. The LED indicators are successfully lit with large-scale conductive thread and the conductive textiles, respectively, indicating the excellent conductivity of large-scale thread and textiles wrapped with Cu-Ag nanonets.

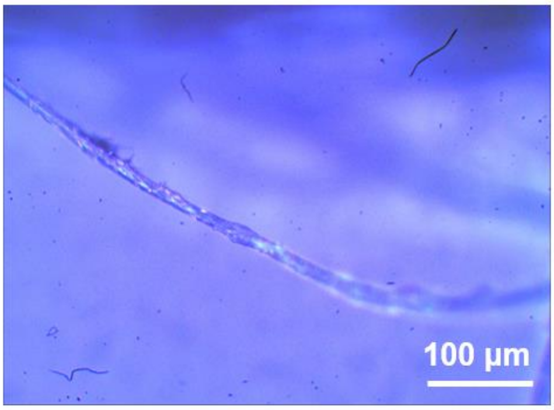

Figure S12. Optical microscope (OM) image of conductive thread.

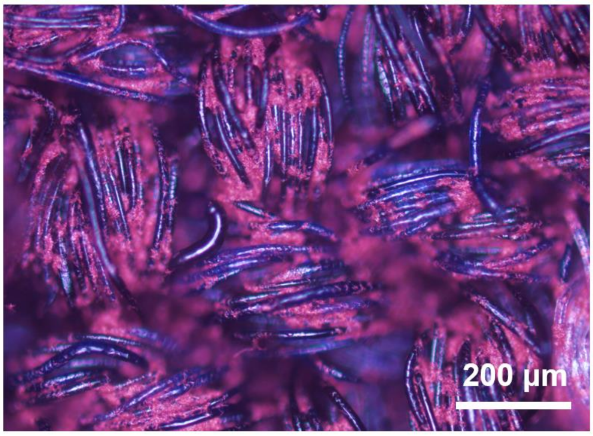

Figure S13. OM image of conductive fabric.

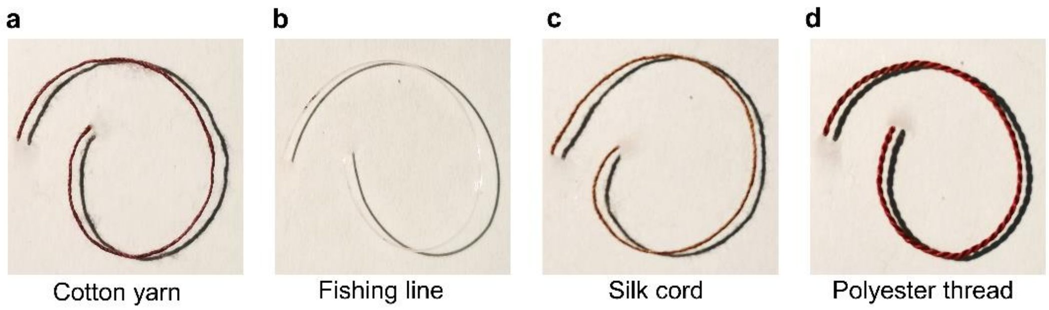

Figure S14. Photographs of different types of curved conductive threads coated by Cu-Ag nanonets with wrapping number of 12.

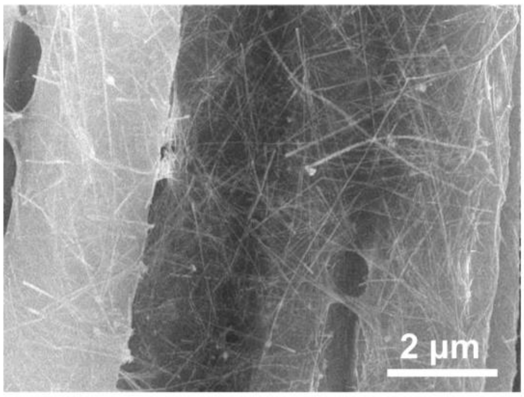

Figure S15. SEM image of the unraveled silk cord fibers coated with Cu-Ag nanonets.

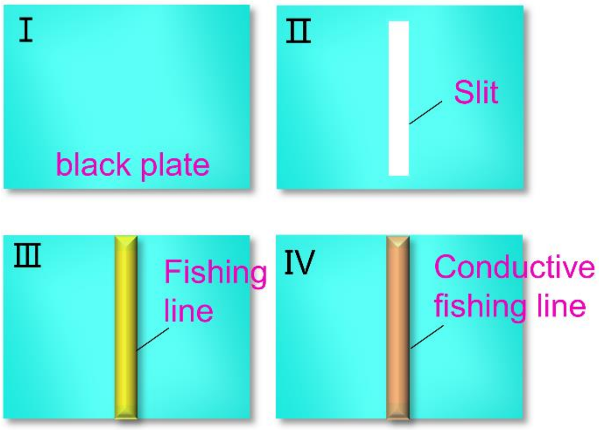

Figure S16. Measuring device and method of transmittance of threads.

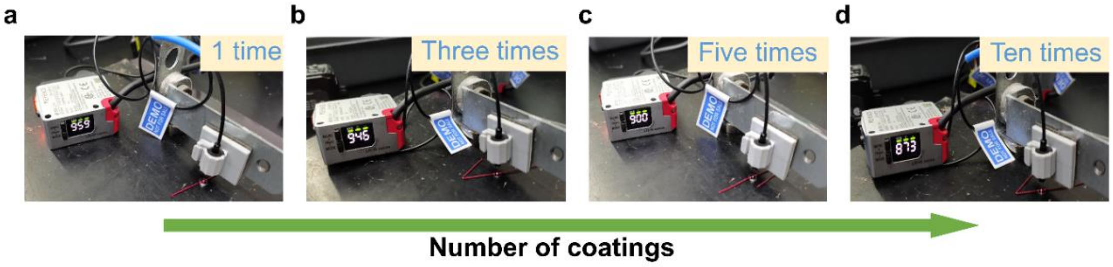

Figure S17. Photos of color similarity tests of conductive silk threads by commercial color sensor.

The color similarity of these conductive threads was analyzed by a commercial color sensor. The color sensor can detect the reflected light of objects and then calculate the intensity ratio of red light, green light and bule light in the reflected light, respectively.

The color similarity is a comprehensive ratio by comparing the RGB intensity ratio of test sample and control sample. Therefore, the color similarity between the two samples (with and without Cu-Ag nanonets wrapping) could be obtained and wrapped threads in various coating numbers were measured, as shown in **Figure S17**. Each sample was tested 5 times for average, as shown in **Figure 3b**. One can see that the color similarity slightly decreases with the increasing wrapping times. Even though, the color similarity can still retain 87% after 10 times wrapping, which indicates the excellent preservation of original color of threads.

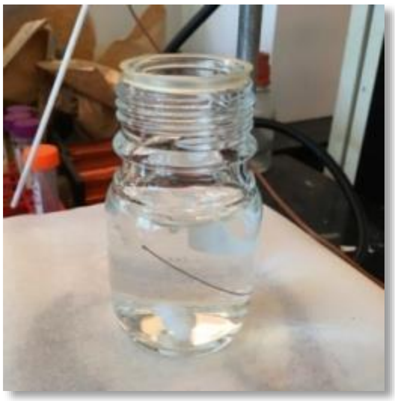

Figure S18. Photograph of stir-washing test.

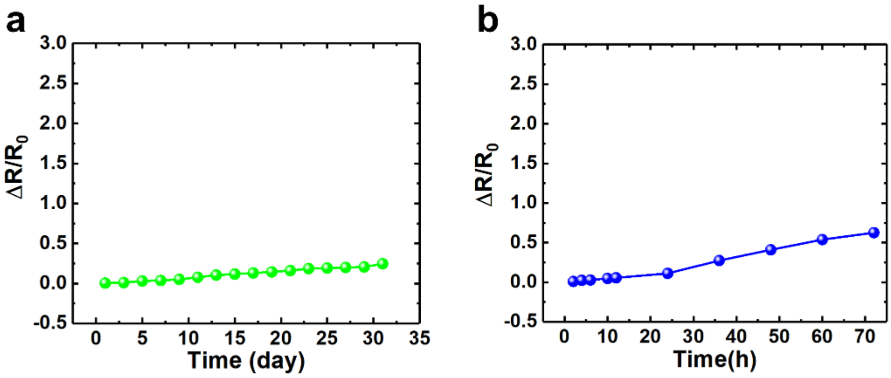

Figure S19. The relative resistance changes of conductive silk cord under room temperature for one month (a), a constant condition with 85 °C and 85% relative humidity for 72 h (b).

The temporal stability of conductive threads was tested under room temperature for

one month and under 85 °C and 85% relative humidity for 72 h, respectively. As shown in **Figure S19**, the resistance of conductive silk cord maintains rather stable in ambient whereas the extreme condition will lead to a slight increase of resistance by 0.6.

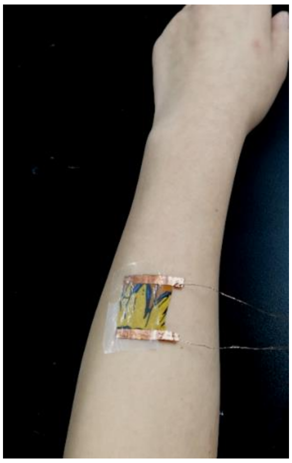

Figure S20. Photograph of temperature sensor on the arm.

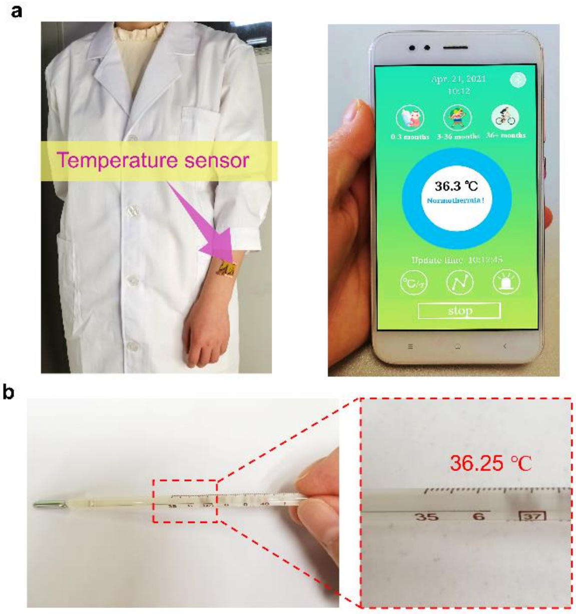

Figure S21. The test results of body temperature on a volunteer by the temperature sensing system made by Cu-Ag nanonets (a) and a mercury thermometer (b) at the same time.

We tested the body temperature on a volunteer by using the flexible cloth sensor with Cu-Ag nanonets and using a conventional mercury thermometer for comparison (**Figure**

S21). As a result, the body temperature tested by our flexible sensing system shows an accuracy of ±0.3 °C, which meets the allowable error of thermometers (*ASTM E1965*).

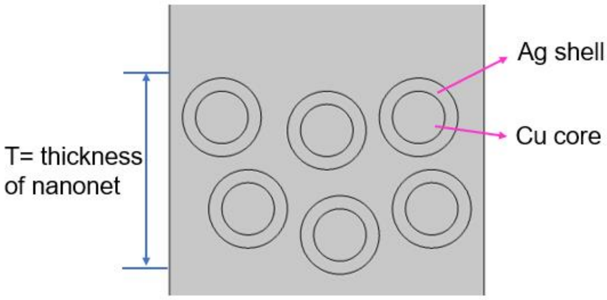

Figure S22. Simulation of Cu-Ag nanonets with 2-D model.

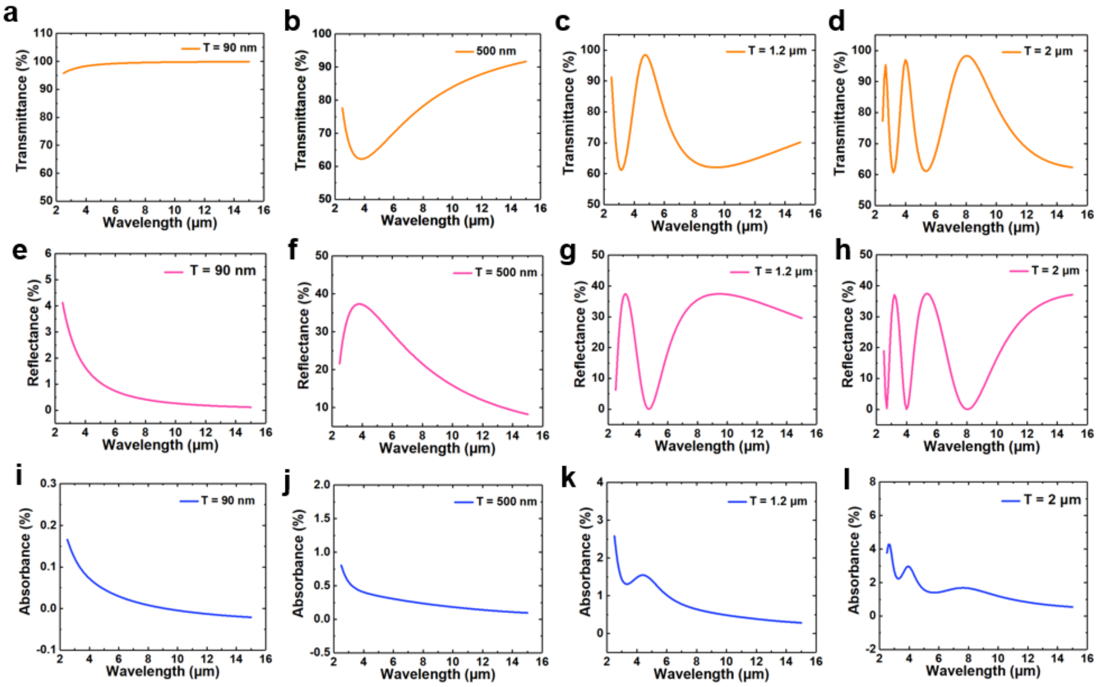

Figure S23. (a-d) Transmittance of Cu-Ag nanonets with thickness of 90 nm, 500 nm, 1.2 μm and 2 μm under mid-infrared wavelength, respectively. (e-h) Reflectance of Cu-Ag nanonets with thickness of 90 nm, 500 nm, 1.2 μm and 2 μm under mid-infrared wavelength, respectively. (i-l) Absorbance of Cu-Ag nanonets with thickness of 90 nm, 500 nm, 1.2 μm and 2 μm under mid-infrared wavelength, respectively.

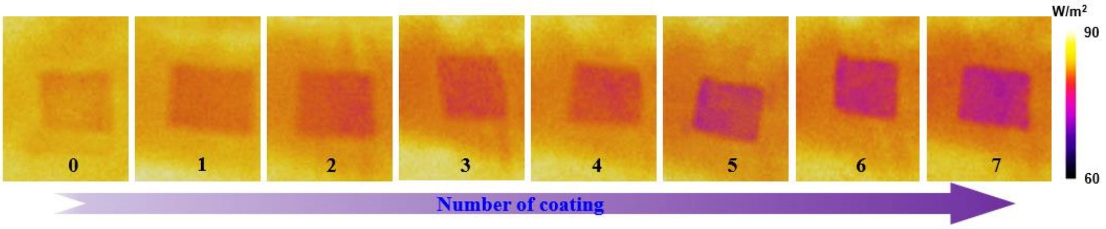

Figure S24. IR thermal images of conductives fabircs with different wrapping number of Cu-Ag nanonets.

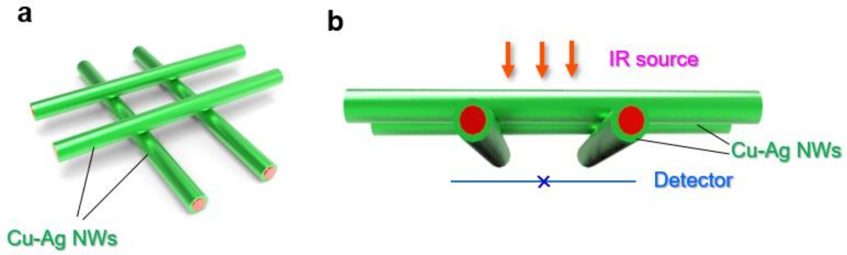

Figure S25. Model structure of crossed Cu-Ag nanonet.

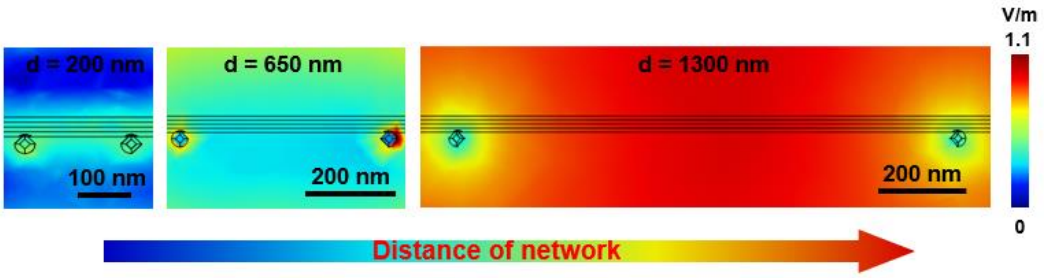

Figure S26. Transverse electric field distribution of Cu-Ag nanonet with different distance of NWs in mid-infrared.

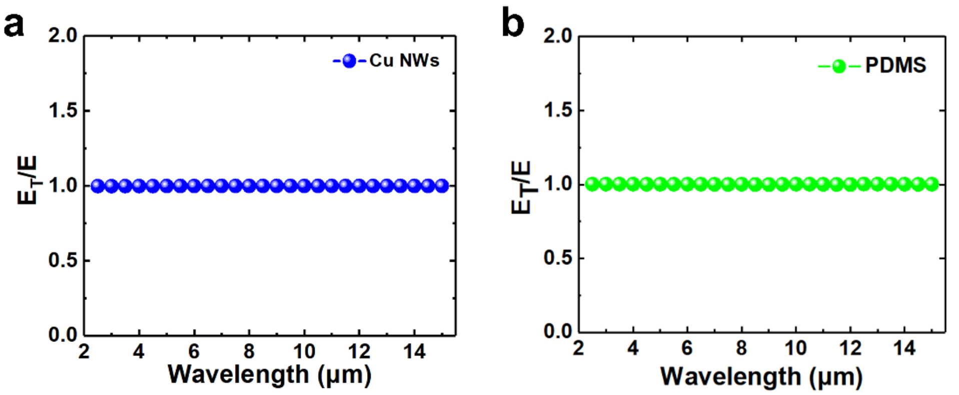

Figure S27. Relative transmission changes of Cu NWs network (a) and PDMS network (b) with distance of 650 nm in mid-infrared.

Table S1 Summary of optoelectronic property of conductive textiles based on silver in recent years.

**Textiles**	**Conductive materials**	**Conductivity**	**Transmittance**	**Techniques**	**Refs.**
Silk yarn	Ag NW/ PEDOT:PSS	320 S/cm	--	Dip-coating	[6]
Cotton yarn+PU fiber	Ag NWs-PDMS	4018 S/cm	--	Dip-costing	[7]
Silk fabric	Ag	480 ohm/sq	--	Electroless plating	[8]
Polyester thread	Ag NWs	15 ohm/cm	--	Dip-coting	[9]
Polyamide fiber	Ag	1.4×10^{-1} ohm/cm	--	Sputter coating	[10]
Polyimide fabric	Ag flakes-PDMS	738 S/cm	--	Self-assembly	[11]
Cotton fabric	Ag NPs, PDA/ PFDT	233.4 S/cm	--	Chemical deposition	[12]
Cotton yarn+PU fiber	Ag NWs	36 ohm/cm	--	Dip-coating	[13]

PU fiber	Ag NWs/SBS	7411 S/m	--	Dip-coating	[14]
Polyester textile	Ag NWs/ graphene	20 ohm/sq	23%	Blade-coating	[15]
**Silk thread**	**Cu-Ag NWs**	**29 ohm/cm**	**80%**	**Dip-coating**	**Our work**

Table S2 Summary of temperature sensor based on conductive textiles in recent years.

**Textiles**	**Conductive materials**	**Measurement range [℃]**	**TCR [$℃^{-1}$]**	**Techniques**	**Refs.**
Cellulose fabric	Copper	40~70	--	Electroless	[16]
Nylon yarn	Cu-Ni wires	5~50	0.04571	Weaving	[17]
Polyester+cotton	PDMS, Ag NPs, graphene	20~100	--	Knitting	[18]
Polyurethane yarn	Ag NPs	25~45	0.0039	Weaving	[19]
--	Nickel wire	20~60	0.0048	Knitting	[20]
**Polyester fabric**	**Cu-Ag NWs**	**34~44**	**0.0589**	**Dip-coating**	**Our work**

Table S3 Summary of textiles with temperature regulating property in recent years.

**Textiles**	**Materials**	**Techniques**	**Temperature regulating [℃]**	**Refs.**
lCotton fabrics	MWCNTs	Ultrasonication	3.9	[21]
Polyester fabric	Polydopamine	Solution method	2.4	[22]
Polyethylene Textiles	Prussian blue	Solution mixing	1.6~1.8	[23]
rPET textiles	Silica aerogels	Solution mixing	2.4~3.5	[24]
Cotton fabrics	TiO_2	Solution method	1.9~3.4	[25]
**Cotton fabrics**	**Cu-Ag NWs**	**Dip-coating**	**0.5~4.5**	**Our work**

Video 1. Video of operating smartphone with normal glove.

Video 2. Video of operating smartphone with touch screen glove made by Cu-Ag nanonets.

Video 3. Video of operating smartphone with touch screen glove made by Cu-Ag nanonets.

Video 4. Video of testing body temperature with sensor fabricated by Cu-Ag nanonets.

References:

[1] H. Wang, C. Wu, Y. Huang, F. Sun, N. Lin, A. M. Soomro, Z. Zhong, X. Yang, X. Chen, J. Kang, ACS Appl. Mater. Interfaces **2016**, 8, 28709.

[2] R. Wang, H. Ruan, J. Alloys Compd. **2016**, 656, 936.

[3] I. E. Stewart, A. R. Rathmell, L. Yan, S. Ye, P. F. Flowers, W. You, B. J. Wiley, Nanoscale **2014**, 6, 5980.

[4] J. Wang, H. Chen, Y. Zhao, Z. Zhong, Y. Tang, G. Liu, X. Feng, F. Xu, X. Chen, D. Cai, J. Kang, ACS Appl. Mater. Interfaces **2020**, 12, 35211.

[5] A. Lim, M.-H. Song, C.-W. Cho, Y.-S. Yun, Applied Sciences **2016**, 6, 378.

[6] B. Hwang, A. Lund, Y. Tian, S. Darabi, C. Muller, ACS Appl. Mater. Interfaces **2020**, 12, 27537.

[7] Y. Cheng, R. Wang, J. Sun, L. Gao, ACS nano **2015**, 9, 3887.

[8] D. Yu, G. Kang, W. Tian, L. Lin, W. Wang, Appl. Surf. Sci. **2015**, 357, 1157.

[9] Y. Atwa, N. Maheshwari, I. A. Goldthorpe, J. Mater. Chem. C **2015**, 3, 3908.

[10] Q. Wei, H. Ye, D. Hou, H. Wang, W. Gao, J. Appl. Polym. Sci. **2006**, 99, 2384.

[11] N. Matsuhisa, M. Kaltenbrunner, T. Yokota, H. Jinno, K. Kuribara, T. Sekitani, T.

Someya, Nat. Commun. **2015**, 6, 1.

[12] B. Niu, S. Yang, T. Hua, X. Tian, M. Koo, Nano Res. **2021**, 14, 1043.

[13] M. Zhao, D. Li, J. Huang, D. Wang, A. Mensah, Q. Wei, J. Mater. Chem. C **2019**, 7, 13468.

[14] S. Chen, S. Liu, P. Wang, H. Liu, L. Liu, J. Mater. Sci. **2017**, 53, 2995.

[15] C. Wu, T. W. Kim, F. Li, T. Guo, ACS Nano **2016**, 10, 6449.

[16] J. Landsiedel, W. Root, N. Aguilo-Aguayo, H. Duelli, T. Bechtold, T. Pham, Sensors (Basel) **2021**, 21.

[17] T. W. Cheung, T. Liu, M. Y. Yao, Y. Tao, H. Lin, L. Li, Text. Res. J. **2021**, 004051752110571.

[18] M. Jung, S. Jeon, J. Bae, RSC Adv. **2018**, 8, 39992.

[19] Q. Li, H. Chen, Z.-Y. Ran, L.-N. Zhang, R.-F. Xiang, X. Wang, X.-M. Tao, X. Ding, Smart Mater. Struct. **2018**, 27, 105017.

[20] M. Husain, R. Kennon, Fibers **2013**, 1, 2.

[21] A. Abbas, Y. Zhao, X. Wang, T. Lin, J Text. Ins. **2013**, 104, 798.

[22] R. Liu, X. Wang, J. Yu, Y. Wang, J. Zhu, Z. Hu, Macromol. Mater. Eng. **2018**, 303, 1700456.

[23] L. Cai, Y. Peng, J. Xu, C. Zhou, C. Zhou, P. Wu, D. Lin, S. Fan, Y. Cui, Joule **2019**, 3, 1478.

[24] D.-Y. Wu, S.-S. Wang, C.-S. Wu, ACS Appl. Polym. Mater. **2021**, 3, 3175.

[25] A. Shams-Nateri, S. Kazemian, N. Piri, J Text. Ins. **2019**, 111, 1223.
